# Mortality following development of breast cancer while using oestrogen or oestrogen plus progestin: a computer record-linkage study

**DOI:** 10.1038/sj.bjc.6602701

**Published:** 2005-08-02

**Authors:** W Chen, D B Petitti, A M Geiger

**Affiliations:** 1 Research and Evaluation Department, Kaiser Permanente Southern California, 100 South Los Robles 2nd Floor, Pasadena, CA 91101, USA

**Keywords:** breast cancer, all-cause mortality, breast cancer mortality, oestrogen, oestrogen plus progestin

## Abstract

The literature on the relationship between breast cancer mortality and postmenopausal oestrogen and combined oestrogen/progestin therapy is seemingly contradictory. This study explored survival after exposure to oestrogen or oestrogen plus progestin at or in the year prior to breast cancer diagnosis. Information on patients first diagnosed with invasive breast cancer between 1993 and 1998 was linked with outpatient pharmacy data from 1992 to 2000. Patients were classified according to use of oestrogen alone or oestrogen plus progestin at or in the year prior to diagnosis. Compared to nonusers, and adjusting for age at diagnosis, race/ethnicity, tumour size and grade, oestrogen receptor status, surgery status, and chemotherapy and hormone therapy for breast cancer treatment, oestrogen plus progestin users had lower all-cause mortality (stage I hazard ratio (HR)=0.69, 95% confidence interval (CI)=0.48–0.99; stage II HR=0.53, 95% CI=0.39–0.72) and breast cancer mortality (stage I HR=0.52, 95% CI=0.26–1.04; stage II HR=0.69, 95% CI=0.48–0.98). Oestrogen users experienced little or no survival benefit for all-cause mortality (stage I HR=1.04, 95% CI=0.77–1.42; stage II HR=0.86, 95% CI=0.65–1.14) or breast cancer mortality (stage I HR=1.23, 95% CI 0.72–2.10; stage II HR=1.01, 95% CI 0.72–1.41). Our findings suggest, relative to nonusers, a lower risk of death from all causes and from breast cancer in patients who were diagnosed with breast cancer while exposed to oestrogen plus progestin, but not in patients exposed to oestrogen only.

Studies have consistently reported a reduced risk of all-cause mortality in hormone users *vs* nonusers (Hunt *et al*, 1990; Henderson *et al*, 1991; Sturgeon *et al*, 1995; Ettinger *et al*, 1996; Sellers *et al*, 1997; Grodstein *et al*, 1997). However, the literature on the relationship between breast cancer mortality and postmenopausal oestrogen and combined oestrogen/progestin therapy is confusing and seemingly contradictory (Bergkvist *et al*, 1989; Hunt *et al*, 1990; Henderson *et al*, 1991; Colditz *et al*, 1995; Sturgeon *et al*, 1995; Persson *et al*, 1996; Willis *et al*, 1996; Grodstein *et al*, 1997; Sellers *et al*, 1997; Sourander *et al*, 1998; Schairer *et al*, 1999; Jernstrom *et al*, 1999; Beral, 2003). A systematic review of postmenopausal hormone replacement therapy and the risk of death from breast cancer found that most studies showed a reduced risk of death from breast cancer in hormone replacement users compared with nonusers, although in some studies the reduction in risk was not statistically significant (Nanda *et al*, 2002).

There is evidence that hormone exposure might increase the incidence of breast cancer (Beral, 2003). However, the effects of hormones used at the time of diagnosis on breast cancer development and on death from breast cancer are not necessarily in the same direction. Comparison of the risk of death from breast cancer for women exposed and not exposed to hormone therapy measures the net effect of hormones on the development of breast cancer and on death from breast cancer given hormone use or nonuse at the time of diagnosis.

This study explored the relationship between survival after breast cancer and exposure to oestrogen or oestrogen plus progestin at or around the time of diagnosis, stratified by cancer stage, and adjusting for other factors associated with survival.

## METHODS

### Design and setting

We conducted a retrospective, computer record-linkage, cohort study among members of a large health maintenance organisation (Kaiser Permanente Southern California, KPSC). The study was approved by the KPSC Institutional Review Board.

### Subjects and data

**Figure 1:**
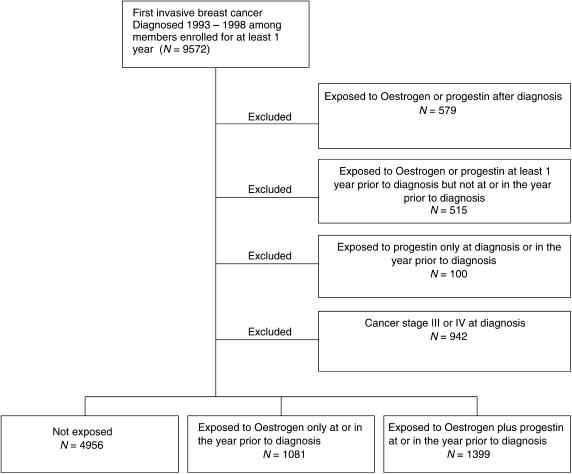
Selection of study subjects.

Using computerised KPSC Cancer Registry records, we identified 9572 patients diagnosed with their first invasive breast cancer between 1 January 1993 and 31 December 1998, who had been continuously enrolled for 1 year prior to diagnosis (Figure 1). Demographic characteristics and information about stage, tumour size and grade, oestrogen receptor (ER) status, progesteron receptor (PR) status, surgery status, and chemotherapy and hormone therapy for first course of treatment was also obtained. The KPSC Cancer Registry is a database that contains information on patients who were diagnosed with cancer at KPSC hospitals or who received at least part of their first course of treatment for cancer at a KPSC hospital. The Registry reports to and follows the requirements of the Surveillance, Epidemiology and End Results (SEER) Program.

We then used KPSC computerised pharmacy data for the period from 1 January 1992 to 31 December 2000 to classify patients according to exposure to oestrogen or progestin during the study period. The pharmacy database contains information on prescriptions filled by health plan members at all KPSC pharmacies. We included prescriptions for oral or transdermal oestrogen or oral progestin alone and excluded prescriptions for drugs marketed as oral contraceptives, as well as vaginal and injectable formulations of oestrogen and progestin.

We ascertained vital status from 1 January 1993 through 31 December 2000 by linking data on our study subjects with computer-stored data on deaths that is derived by probabilistic matching of KPSC membership data with California death files. To assure accuracy in designation of vital status, uncertain matches defined by a linkage weight less than 10 were manually examined and validated as true matches by visual inspection. Deaths with the 9th revision of International Classification of Disease (ICD) code 174 or the 10th revision of the ICD code C50 as the underlying cause of death were considered deaths from breast cancer.

As we were interested in the impact of exposure to oestrogen with or without progestin at or in the year prior to diagnosis, we excluded from the analysis 579 patients exposed to oestrogen or progestin after diagnosis; 515 past users of oestrogen with or without progestin; and 100 users of progestin only at or in the year prior to diagnosis (Figure 1). Finally, we excluded 942 patients with TNM stage III or IV cancer due to their poorer prognosis relative to earlier stage breast cancers. There remained 7436 patients with stage 0, I or II breast cancer. These included 1081 patients who exposed to oestrogen only and 1399 patients who exposed to both oestrogen and progestin at or in the year prior to diagnosis, and 4956 nonusers.

### Analysis

As stage at diagnosis may be associated with exposure to oestrogen and progestin and with survival, we stratified all analyses by stage. We first performed a univariate analysis examining characteristics of patients by hormone status. To test the statistical significance of differences between groups, we used the *χ*^2^ test or Fisher's exact test for categorical variables and analysis of variance (ANOVA) for continuous variables.

As the impact of oestrogen and progestin use might differ between all-cause mortality and breast cancer mortality, both outcomes were examined. Patient follow-up started from the breast cancer diagnosis and ended with an outcome (either all-cause death or breast cancer death) or when censoring occurred, on the earliest of the following dates: health plan disenrollment; end of study (31 December 2000); and nonbreast cancer death when the outcome was breast cancer mortality. The Cox proportional hazards model was used to estimate the hazard ratio for mortality in relation to oestrogen and progestin exposure with nonhormone users as the referent. Crude, age-adjusted, and age plus other confounder-adjusted hazard ratios (HR) were calculated along with their 95% confidence intervals (CI).

Adjusted survival curves were generated from the Cox models by setting the covariates at their means. The covariates included in these models were age at diagnosis, race/ethnicity, tumour size and grade, ER status, surgery status, and chemotherapy and hormone therapy for first course of treatment.

All analyses were conducted with SAS software (version 8.02 for Windows, SAS Institute, Cary, NC, USA).

## RESULTS

Among the 7436 eligible patients with breast cancer, 848 (11. 4%) died during the study period. Of these deaths, 421 (5.7% of all patients; 49.6% of all deaths) were attributed to breast cancer. Only 10 patients diagnosed with stage 0 died of breast cancer (Table 1) and therefore further data are not shown for stage 0 patients. For patients with stage I or II cancer, all-cause mortality was about 8% in patients exposed to oestrogen plus progestin, 13–15% in patients exposed to oestrogen only, and 18–19% in nonhormone users. A similar pattern was found for breast cancer mortality although the stage-specific breast cancer mortality rates differed between stage I and II cancer patients.

Across stages I and II, exposure to oestrogen and progestin at the time of diagnosis varied by age, race/ethnicity, and PR status (Tables 2A and B). Oestrogen receptor status only differed by oestrogen and progestin exposure status in stage II cancer patients (*P*=0.010). Follow-up time was longer among nonhormone users for both stage I and II cancers (*P*<0.01). Among patients with stage II cancer, nonhormone users presented with larger tumours (*P*=0.021); nonhormone users were more likely to present with poorly differentiated tumours for both stages (*P*<0.001). In both stage I and II patients, those who were exposed to hormones at the time of or near breast cancer diagnosis were more likely to be treated with hormones for their breast cancer after diagnosis (*P*<0.001). During the time period in which patients in this study were diagnosed with breast cancer (1993–1998), the most common hormone therapy for breast cancer treatment in use was tamoxifen.

In both stage I and II patients, use of oestrogen plus progestin at the time of or near breast cancer diagnosis significantly reduced risk of all-cause mortality (Table 3A and B). After adjusting for age at diagnosis, race/ethnicity, tumour size and grade, ER status, surgery status, and chemotherapy and hormone therapy for breast cancer treatment, the HR of all-cause mortality for oestrogen plus progestin users *vs* nonusers were 0.69 (95% CI 0.48–0.99) for stage I and 0.53 (95% CI 0.39–0.73) for stage II cancer patients. Breast cancer mortality was reduced in stage II patients (HR 0.69, 95% CI 0.48–0.98). In stage I patients the risk reduction was almost 50% but it was not statistically significant (HR 0.52, 95% CI 0.26–1.04).

In both stage I and II patients, use of oestrogen alone at the time of or near breast cancer diagnosis did not increase or decrease all-cause or breast cancer mortality (Table 3A and B). After adjusting for age at diagnosis, race/ethnicity, tumour size and grade, ER status, surgery status, and chemotherapy and hormone therapy for breast cancer treatment, the HR of all-cause mortality for oestrogen only users *vs* nonusers were 1.04 (95% CI 0.77–1.42) for stage I and 0.86 (95% CI 0.65–1.14) for stage II cancer patients. The corresponding HR for breast cancer mortality were 1.23 (95% CI 0.72–2.10) and 1.01 (95% CI 0.72–1.41).

**Figure 2:**
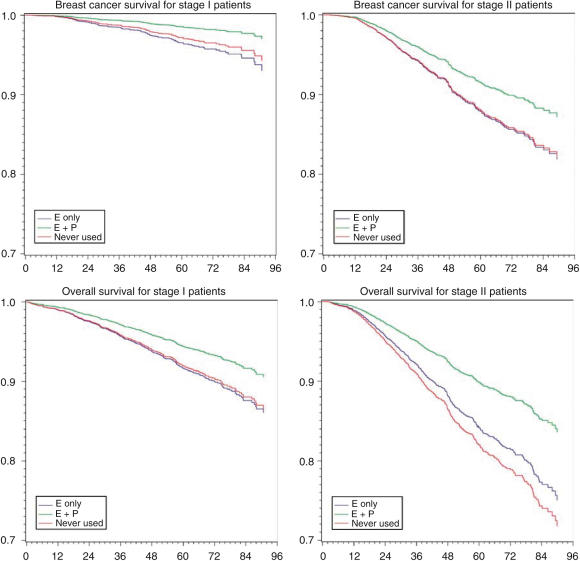
Adjusted survival curves by hormone exposure status. *Y*-axis label, survival function estimate. *X*-axis label, months after breast cancer diagnosis. All survival curves were adjusted for age at diagnosis, race/ethnicity, tumour size and grade, oestrogen receptor status, surgery status, and chemotherapy and hormone therapy for breast cancer treatment.

Figure 2 shows that although the overall survival varied by cancer stage, the improvement in survival among oestrogen plus progestin users compared to nonusers remains consistent across both cancer stages for both all-cause mortality and breast cancer mortality.

We repeated the analyses excluding patients aged 55 years or younger because these women might have been premenopausal. The results were similar (data not shown).

## DISCUSSION

This observational study of patients with breast cancer assessed mortality for all causes and for breast cancer in relation to oestrogen and progestin exposure at or in the year prior to breast cancer diagnosis. Our results are mixed. They show a lower mortality from all causes and from breast cancer in patients who were diagnosed with breast cancer while exposed to oestrogen plus progestin. Neither all-cause nor breast cancer mortality was decreased in the patients with breast cancer who were exposed to oestrogen alone.

Several other studies have reported lower all-cause or breast cancer mortality (Bergkvist *et al*, 1989; Hunt *et al*, 1990; Sturgeon *et al*, 1995; Willis *et al*, 1996; Persson *et al*, 1996; Sellers *et al*, 1997; Jernstrom *et al*, 1999; Schairer *et al*, 1999) in hormone users compared with nonusers, while others have found no significant differences in breast cancer mortality in hormone users compared with nonusers (Hunt *et al*, 1990; Henderson *et al*, 1991; Grodstein *et al*, 1997; Sourander *et al*, 1998). The Million Women Study (MWS) reported an increased risk (RR 1.22, 95% CI 1.05–1.41) of breast cancer mortality comparing current-users with never-users (Beral, 2003). However, Bundred and Morris (2003) analysed summary data from the MWS publication and showed that the crude relative risk of breast cancer mortality was 0.725 for current users *vs* never-users in the subgroup of women diagnosed with breast cancer. Nevertheless, the lack of information on stage at diagnosis and treatment makes the interpretation of these results difficult. In 1995, the Nurses' Health Study reported a relative risk of death from breast cancer of 1.14 (95% CI 0.85–1.51) for current hormone users and a risk of 1.45 (95% CI 1.01–2.09) for hormone users of five or more years (Colditz *et al*, 1995). However, in 1997, after 2-years of additional data and with adjustment of additional covariates, the adjusted relative risk of mortality due to breast cancer was 0.76 (95% CI 0.56–1.02) among current hormone users compared with nonusers (Grodstein *et al*, 1997).

Few studies have directly assessed prognosis in breast cancer that develops in women using hormones at the time of diagnosis compared with those who were not using hormones at diagnosis. The studies close in design to our study are those of Bergkvist *et al*, Schairer *et al*, and Jernstrom *et al*, who also examined mortality in women diagnosed with breast cancer in hormone users with mortality in women diagnosed with breast cancer who were not hormone users (Bergkvist *et al*, 1989; Schairer *et al*, 1999; Jernstrom *et al*, 1999). Of these, Bergkvist *et al*'s study is closest in design to ours in that breast cancer patients who were exposed to oestrogen at different time periods in relation to diagnosis were compared with breast cancer patients who had had no previous oestrogen therapy. Oestrogen users had lower all-cause mortality, but the effect was more pronounced in recent users and users who had been discontinued within a year prior to diagnosis (Bergkvist *et al*, 1989).

Only a few studies have examined the association between hormone type and breast cancer mortality. The results are mixed (Bergkvist *et al*, 1989; Persson *et al*, 1996). Bergkvist reported a greater reduction in breast cancer mortality in users of oestrogen plus progestin than in user of oestrogen only (relative hazard 0.87 for oestrogen only users and 0.50 for oestrogen plus progestin). Persson *et al* (1996) reported that women prescribed a fixed dose oestrogen–progestin combination consisting of oestradiol and levonorgestrel had smaller decrease in the relative risk of breast cancer mortality than women prescribed oestrogen only. However, as noted above, in Persson's study the standardised breast cancer mortality ratios were lower than 1.0 in all hormone use groups.

Several observational studies have reported that breast cancers in hormone users are diagnosed earlier and have histologic and other features that are associated with a better prognosis (Holli *et al*, 1998; Sacchini *et al*, 2002; Daling *et al*, 2003; Pappo *et al*, 2004) However, results from the Women's Health Initiative (WHI), the only randomised controlled trial of hormone use, showed that hormone users were diagnosed with larger tumours and more likely to have positive lymph nodes (Chlebowski *et al*, 2003). The study was underpowered to access the effect on grade, histology and hormone receptor status. A recent systematic review of 25 studies that evaluated breast cancer risk factors and prognostic indicators concluded that the WHI study is in contradiction to most published observational studies (Antonie *et al*, 2004). Our results also appear to contradict the WHI results. This discrepancy might be due to biases in the observational studies including lack of adjustment for important confounding factors. It might also be due to the highly unrepresentative nature of WHI enrollees in terms of age and time since menopause.

Understanding of the mechanisms underlying breast tumour aggressiveness, prognosis and metastatic potential is incomplete. Progesterone receptor is a prognostic marker in breast cancer. Two functional progesterone receptor isoforms, PR-A and PR-B, have been identified. Recent studies in animals have shown the ratio of the isoforms affects tumour phenotype (Jacobsen *et al*, 2003, 2005) and perhaps response to endocrine therapy (Sartorius *et al*, 2003; Hopp *et al*, 2004; Wu *et al*, 2004). It is possible that exogenous synthetic progestins alter the balance between PR-A and PR-B in malignant breast tumours, thus affecting tumour aggressiveness. This is admittedly highly speculative.

Our study has some strengths. First, the study involved a very large number of cases of breast cancer, permitting estimation of mortality stratified by cancer stage. Second, our study was able to classify patients with breast cancer according to the use of oestrogen alone or oestrogen plus progestin within each stage. Third, our study was able to assess mortality risk in relation to oestrogen and progestin exposure at the time or near breast cancer diagnosis with large numbers. Finally, we were able to examine both all-cause and breast cancer mortality.

Our study has several limitations. First, it is based on computer record linkage. Patients were classified as exposed to oestrogen and progestin based on having filled a prescription but it is possible that they did not actually consume the drug. Also, for health plan members who did not have drug coverage for oestrogen and progestin (about 8%) and had to pay full member rate, the information about exposure to hormones may be incomplete. Second, in spite of the large number of breast cancer cases, breast cancer mortality in stage 0 cancer patients was too low to permit estimation of the risk of breast cancer mortality in relation to hormone exposure in this group. Third, it is possible that death due to causes other than breast cancer were assigned to breast cancer because of a prior diagnosis of breast cancer. Fourth, only 30 and 67% of deaths were attributable to breast cancer in stage I and II patients, respectively. If hormone users were healthier for reasons other than hormone use, all-cause mortality would be biased due to the ‘healthy user’ and/or ‘healthy complier’ effect (Barrett-Connor, 1991; Petitti, 1994). Fifth, hormone users, particularly if they were healthier, might have received additional treatments beyond their first course of therapy. These treatments were not available in the KPSC Cancer Registry and thus could not be incorporated into the analysis. However, there is no reason to expect these limitations would be different between oestrogen only users and users of oestrogen plus progestin.

Given the limitations, the study results should be interpreted cautiously. Other large databases of computer-stored prescription information are available and could be used to attempt to replicate or refute our observations.

## Figures and Tables

**Table 1:** Distribution of hormone use and death by stage at diagnosis

**Stage**	**Hormone exposure**	**No. of patients (*N*=7436)**	***N* (%) death from all causes**	***N* (%) death from breast cancer**
0	Oestrogen only	142	5 (3.5)	1 (0.7)
	Oestrogen plus progestin	180	3 (1.7)	1 (0.6)
	Neither	628	38 (6.1)	3 (0.5)
	Total	950	46 (4.8)	5 (0.5)
				
I	Oestrogen only	532	50 (14.7)	17 (3.2)
	Oestrogen plus progestin	678	34 (8.5)	9 (1.3)
	Neither	2259	244 (17.8)	73 (3.2)
	Total	3469	328 (9.5)	99 (2.9)
				
II	Oestrogen only	407	60 (12.7)	41 (10.1)
	Oestrogen plus progestin	541	46 (8.3)	36 (6.7)
	Neither	2069	368 (18.5)	240 (11.6)
	Total	3017	474 (15.7)	317 (10.5)

**Table 2:** Characteristics of patients with (A) stage I and (B) stage II breast cancer by hormone exposure status at the time of breast cancer diagnosis

**(A)**				
	**Hormone exposure status**			
	**Oestrogen only (*N*=532)**	**Oestrogen plus progestin (*N*=678)**	**Neither (*N*=2259)**	***P***
Mean (s.d.) age at diagnosis in years	63.4 (9.6)	61.2 (8.0)	61.0 (13.9)	<0.001
				
Mean (s.d.) month of follow-up	50.2 (21.3)	50.1 (21.7)	54.0 (23.4)	<0.001
				
*Race/ethnicity* (%)				<0.001
African American	6.9	5.6	13.0	
Asian	4.3	6.6	7.3	
Latino	5.1	5.2	8.9	
White	83.1	81.9	70.0	
Other/unknown	0.6	0.7	0.8	
				
*Tumour size* (%)				0.130
<1.0 cm	38.7	40.0	37.5	
⩾1.0 cm	53.8	54.1	57.4	
Unknown	7.5	5.9	5.1	
				
*Tumour grade* (%)				<0.001
Well differentiated	26.9	29.9	23.0	
Moderately differentiated	36.5	41.5	39.4	
Poorly differentiated	18.4	16.8	21.4	
Unknown	18.2	11.8	16.2	
				
*Oestrogen receptor* (%)				0.417
Positive	66.4	65.8	62.7	
Negative	14.5	14.3	16.1	
Other/not done/unknown	19.2	19.9	21.2	
				
*Progesterone receptor* (%)				<0.001
Positive	51.5	44.8	45.9	
Negative	15.2	12.6	18.2	
Other/not done/unknown	33.3	42.6	35.9	
				
*Hormone therapy for breast cancer treatment*^a^ (%)				<0.001
Yes	58.3	56.6	46.8	
No	41.7	43.4	53.2	
				
*Chemotherapy* (%)				<0.001
Yes	12.4	12.4	19.6	
None/other/unknown	87.6	87.6	80.4	
				
*Surgery* (%)				0.007
Lumpectomy	60.5	63.9	56.9	
Mastectomy	38.7	35.4	41.5	
None/other/unknown	0.8	0.7	1.6	
				

**(B)**				
	**Oestrogen only (*N*=407)**	**Oestrogen plus progestin (*N*=541)**	**Neither (*N*=2069)**	***P***
Mean (s.d.) age at diagnosis in years	61.5 (9.3)	59.5 (7.9)	55.9 (14.5)	<0.001
				
Mean (s.d.) month of follow-up	47.1 (20.7)	47.9 (20.9)	50.3 (23.0)	0.007
				

**(B)**				
	**Hormone exposure status**			
	**Oestrogen only (*N*=407)**	**Oestrogen plus progestin (*N*=541)**	**Neither (*N*=2069)**	***P***
*Race/ethnicity* (%)				<0.001
African American	10.3	7.8	17.1	
Asian	4.4	6.6	8.8	
Latino	8.1	7.6	12.6	
White	76.7	78.0	61.2	
Other/unknown	0.5	0.0	0.3	
				
*Tumour size* (%)				0.021
<2.0 cm	37.4	35.9	29.9	
2.0–4.9 cm	53.8	53.8	59.5	
⩾5.0 cm	3.9	4.8	5.5	
Unknown	4.9	5.5	5.1	
				
*Tumour grade*				0.001
Well differentiated	11.8	15.0	9.8	
Moderately differentiated	38.3	40.5	37.9	
Poorly differentiated	35.4	33.8	41.3	
Unknown	14.5	10.7	11.0	
				
*Oestrogen receptor* (%)				0.010
Positive	62.2	63.0	56.8	
Negative	24.3	19.6	25.2	
Other/not done/unknown	13.5	17.4	18.0	
				
*Progesterone receptor* (%)				0.028
Positive	47.2	43.4	42.2	
Negative	24.8	20.0	24.2	
Other/not done/unknown	28.0	36.6	33.6	
				
*Hormone therapy for breast cancer treatment*^a^ (%)				<0.001
Yes	52.3	54.5	43.3	
No/unknown	47.7	45.5	56.7	
				
*Chemotherapy* (%)				0.172
Yes	60.0	61.6	64.3	
None/other/unknown	40.0	38.4	35.7	
				
*Surgery* (%)				0.167
Lumpectomy	41.8	41.0	37.4	
Mastectomy	57.0	58.4	61.2	
None/other/unknown	1.2	0.6	1.4	

a During the study period, the most common hormone therapy in use for breast cancer treatment was tamoxifen.

**Table 3:** Crude and adjusted hazard ratios (HR) and 95% confidence intervals (CI) for patients with (A) stage I and (B) stage II breast cancer according to hormone exposure status at the time of diagnosis

**Adjustment**	**Hormone exposure**	**All-cause mortality HR (95% CI)**	**Mortality from breast cancer HR (95% CI)**
**(A)**			
None	Oestrogen only	0.95 (0.70–1.29)	1.10 (0.65–1.87)
	Oestrogen plus progestin	0.51 (0.36–0.73)	0.46 (0.23–0.91)
	Neither	1.00 (referent)	1.00 (referent)
			
Age only	Oestrogen only	1.00 (0.74–1.36)	1.09 (0.64–1.85)
	Oestrogen plus progestin	0.63 (0.44–0.90)	0.46 (0.23–0.92)
	Neither	1.00 (referent)	1.00 (referent)
			
Multivariable^a^	Oestrogen only	1.04 (0.77–1.42)	1.23 (0.72–2.10)
	Oestrogen plus progestin	0.69 (0.48–0.99)	0.52 (0.26–1.04)
	Neither	1.00 (referent)	1.00 (referent)
			
**(B)**			
None	Oestrogen only	0.90 (0.68–1.18)	0.94 (0.67–1.31)
	Oestrogen plus progestin	0.51 (0.38–0.69)	0.61 (0.43–0.87)
	Neither	1.00 (referent)	1.00 (referent)
			
			
Age only	Oestrogen only	0.82 (0.63–1.08)	0.96 (0.69–1.34)
	Oestrogen plus progestin	0.49 (0.36–0.66)	0.62 (0.44–0.88)
	Neither	1.00 (referent)	1.00 (referent)
			
			
Multivariable^a^	Oestrogen only	0.86 (0.65–1.14)	1.01 (0.72–1.41)
	Oestrogen plus progestin	0.53 (0.39–0.73)	0.69 (0.48–0.98)
	Neither	1.00 (referent)	1.00 (referent)

a Adjusted for age at diagnosis, race/ethnicity, tumour size and grade, oestrogen receptor status, surgery status, and chemotherapy and hormone therapy for breast cancer treatment.
